# Surgeon-General of the United States

**Published:** 1861-07

**Authors:** 


SURGEON-GENERAL OF THE UNITED STATES.—Dr. Finley, of Pennsylvania, succeeds Dr. Lawson, recently deceased, to this important office.

Dr. Gibbs, of South Carolina, is Surgeon-in-chief of the Confederate Army.

Dr. Eve, of Nashville, holds the office of Surgeon-general to the Volunteer forces of Tennessee.

